# Proteomic peptide profiling for preemptive diagnosis of acute graft-versus-host disease after allogeneic stem cell transplantation

**DOI:** 10.1038/leu.2013.210

**Published:** 2013-07-11

**Authors:** E M Weissinger, J Metzger, C Dobbelstein, D Wolff, M Schleuning, Z Kuzmina, H Greinix, A M Dickinson, W Mullen, H Kreipe, I Hamwi, M Morgan, A Krons, I Tchebotarenko, D Ihlenburg-Schwarz, E Dammann, M Collin, S Ehrlich, H Diedrich, M Stadler, M Eder, E Holler, H Mischak, J Krauter, A Ganser

**Affiliations:** 1 Department of Hematology, Hemostasis, Oncology and Stem Cell Transplantation, Laboratory of Transplantation Biology, Hannover Medical School, Hannover, Germany; 2 Mosaiques Diagnostics GmbH, Hannover, Germany; 3 Deptartment of Hematology and Oncology, University of Regensburg, Regensburg, Germany; 4 Deutsche Klinik für Diagnostik, Wiesbaden, Germany; 5 Department of Internal Medicine I, Medical University of Vienna, Vienna, Austria; 6 Department of Hematology, University of Newcastle upon Tyne, Newcastle upon Tyne, UK; 7 Biomarkers and Systems Medicine group, University of Glasgow, Glasgow, UK; 8 Department of Pathology, Hannover Medical School, Hannover, Germany; 9 Institute of Experimental Hematology, Hannover Medical School, Hannover, Germany

**Keywords:** hematopoietic stem cell transplantation, graft-versus-host disease, proteomics, capillary electrophoresis, mass spectrometry, Proteomics, Graft-versus-host disease, Diagnosis, Cell transplantation

## Abstract

Allogeneic hematopoietic stem cell transplantation is one curative treatment for hematological malignancies, but is compromised by life-threatening complications, such as severe acute graft-versus-host disease (aGvHD). Prediction of severe aGvHD as early as possible is crucial to allow timely initiation of treatment. Here we report on a multicentre validation of an aGvHD-specific urinary proteomic classifier (aGvHD_MS17) in 423 patients. Samples (*n*=1106) were collected prospectively between day +7 and day +130 and analyzed using capillary electrophoresis coupled on-line to mass spectrometry. Integration of aGvHD_MS17 analysis with demographic and clinical variables using a logistic regression model led to correct classification of patients developing severe aGvHD 14 days before any clinical signs with 82.4% sensitivity and 77.3% specificity. Multivariate regression analysis showed that aGvHD_MS17 positivity was the only strong predictor for aGvHD grade III or IV (*P*<0.0001). The classifier consists of 17 peptides derived from albumin, β2-microglobulin, CD99, fibronectin and various collagen α-chains, indicating inflammation, activation of T cells and changes in the extracellular matrix as early signs of GvHD-induced organ damage. This study is currently the largest demonstration of accurate and investigator-independent prediction of patients at risk for severe aGvHD, thus allowing preemptive therapy based on proteomic profiling.

**Supplementary information:**

The online version of this article (doi:10.1038/leu.2013.210) contains supplementary material, which is available to authorized users.

## Introduction

Allogeneic hematopoietic stem cell transplantation (allo-HSCT) is one curative treatment for adult patients with high-risk acute leukemia or severe hematopoietic failure syndromes. Overall survival is about 40% (range 25–62%) for leukemia patients depending on primary disease, stage, conditioning regimens^1, 2^ and risk groups (range: 25% (high-risk leukemia) to 62% (good-risk leukemia)),^3^ and about 90% for hematopoietic failure syndrome patients.^4, 5, 6^ However, allo-HSCT is associated with major complications, such as severe acute graft-versus-host disease (aGvHD) and infections.^7, 8, 9^ Differential diagnosis of aGvHD from treatment-related toxicities can be difficult and is mainly made according to clinical symptoms and biopsies. Thus, a method is urgently needed to diagnose early onset of aGvHD and to identify patients at risk of developing severe GvHD in an observer-independent, unbiased fashion. Depending on the type of transplantation, patient age, the immunosuppressive prophylaxis and the underlying disorders, 35–85% of transplanted patients develop aGvHD.^7, 10, 11^ First-line therapy of aGvHD consists of steroids resulting in a response rate of about 70% for patients with aGvHD grade I or II without significant increase of mortality.^10^ In contrast, patients developing aGvHD grades III or IV have a mortality risk of about 80–90% due to aGvHD-specific organ dysfunction or concomitant infections.^12^ Recently, proteome analysis of body fluids using capillary electrophoresis (CE) coupled on-line to mass spectrometry (MS) to define differentially excreted peptides has been shown to be a powerful new diagnostic tool in a variety of diseases and is broadly applicable.^13, 14, 15, 16, 17^ CE-MS has been applied to identify biomarkers for early detection of aGvHD in patients undergoing allo-HSCT since 2003.^18, 19, 20^ We employed these biomarkers to generate an aGvHD-specific classifier, aGvHD_MS17, that allowed distinction of patients with severe aGvHD (grades III and IV) from those who never developed aGvHD, patients with low or moderate aGvHD (grades I and II) and patients with chronic GvHD (cGvHD) after allo-HSCT. In the present study, we prospectively evaluated the predictive value of aGvHD_MS17 in 423 patients who were enrolled in one of five participating transplant centers and who were transplanted between 2005 and 2010. Results obtained from aGvHD_MS17 analysis were superior to results for other biomarkers previously described for prediction or diagnosis of aGvHD, such as loss of serum albumin,^21^ C-reactive protein^22^ and plasma biomarkers.^23^ This report represents the largest study using proteomics in patient assessment. Our results demonstrate the predictive value, clinical usefulness and applicability of this novel diagnostic tool in post-HSCT surveillance.

## Patients and methods

### Patients

Prospectively collected midstream urine samples from 429 patients undergoing allo-HSCT between 2005 and 2010 were obtained after informed consent (ethic protocol number 3790). Six patients died before engraftment and were excluded from further analysis. A summary of all clinical data is shown in Tables 1a–c. Of 423 recipients, 242 were male, 80 of those were transplanted from female donors and for 16 no information on donor gender was available. Immunosuppressive antibodies were administered to 308 (72%) patients. For 17 patients, no information regarding antibody treatment was available. Diagnosis of aGvHD was based on clinical criteria^24^ and on histopathology of biopsies, if available (Table 1b and c). Diagnosis of cGvHD followed criteria established in the cGvHD diagnosis and treatment consensus conferences 2007 and 2009 (ref. 25) and adapted to European needs.^26^ Incidence and severity of acute GvHD and information on biopsies are summarized in Tables 1b and c. Twenty-five patients died before day +100, six had aGvHD as cause of death. All patients were examined daily during hospitalization and weekly thereafter for the first 130 days post allo-HSCT. Clinical aGvHD was assessed according to the aGvHD score from grade 0 (no sign of GvHD) to IV.^24^

**Table 1a:** Clinical characteristics of all patientspa

	*Prospective (*n*=423)*
Age	49 (17–71)
*Disease*	
Acute (AML, ALL and sAML)	268
Chronic (MDS, MPS, CML and CLL)	78
Lymphoma ( MM, NHL and HD)	68
Nonmalignant (AA and PNH)	9
*Status*	
CR 1/CP 1	129
CR 2 or higher	48
no CR (untreated, relapse and refractory)	217
No status (AA, no information)	29
*Conditioning*	
Myeloablative	134
RIC	285
Unknown	4
*Graft*	
PBSC	379
BM	39
CB	5
*GvHD prophylaxis*	
CSA/MTX	197
CSA/MMF	189
TCD	6
Other	29
None	2
*Immunosuppressive antibodies*	
ATG, thymoglobulin	308
None^a^	98
*Donor*	
Related	92
Unrelated	331
*HLA match*	
Matched	333
Mismatched	90
*Gender*	
Female/male	181/242
Male recipient/female donor^b^	80
Engraftment failure	None
Death before day +100	25

Abbreviations: AA, severe or very severe aplastic anemia; ALL, acute lymphatic leukemia; AML, acute myeloid leukemia; ATG, antithymocyte globulin; BM, bone marrow; CB, cord blood; CLL, chronic lymphatic leukemia; CML, chronic myeloid leukemia; CP, chronic phase; CR, complete remission; CSA, cyclosporine A; HD, Hodgkin’s disease; HLA, human leukocyte antigen; MDS, myelodysplastic; MM, multiple myeloma; MMF, mycophenolate mofetil; MPS, myeloproliferative syndrome; MTX, methotrexate; NHL, Non-Hodgkin’s lymphoma; PBSC, peripheral blood stem cell; PNH, paroxysmal nocturnal hematuria; RIC, reduced intensity conditioning; sAML, secondary AML; TCD, T-cell depletion (*ex vivo*: CD34-selection); other, MMF, tacrolimus (FK506), steroids or different combinations; None, no additional GvHD prophylaxis (*ex vivo* T-cell depletion or syngeneic donors).

Sixty-three percent of the patients were transplanted for acute leukemia (*n*=268), 78 for chronic malignant disease, 68 for lymphomas and 9 for hematopoietic failure syndromes. At the time of transplantation, 51% (*n*=217) were not in CR, and for 20 patients information on disease status before transplantation was not available. Myeloablative conditioning (*n*=134; 31%) consisted of total body irradiation (TBI) (12 Gy) or busulfan (16 mg/kg body weight (BW)) in combination with cyclophosphamide (120 mg/kg BW). RIC protocols (*n*=285; 67%) were administered because of high-risk leukemia, >5% blasts in the BM, co-morbidities not allowing standard conditioning or because of age (>60 years). The ‘Flamsa-protocol’ was the most frequently applied RIC, and it consisted of fludarabine, high-dose cytarabine, amsacrine, followed by 4 Gy TBI and cyclophosphamide and immunosuppressive antibodies as an additional aGvHD prophylaxis. The majority of the patients received PBSCs (*n*=379; 89%), 39 received BM and 5 were transplanted with double CB transplantation. aGvHD prophylaxis consisted of CSA and MTX (*n*=197; 46.5%) or MMF (*n*=189; 44.6%); or other combinations (*n*=29); *ex vivo* CD34-enrichment (TCD) without additional GvHD prophylaxis (*n*=6), or no GvHD prophylaxis for other reasons (*n*=2). Immunosuppressive antibodies were administered before HSCT (day −3 to −1) to 308 patients (72%). ATG (Fresenius, Munich, Germany) was administered at 20 mg/kg BW per day for matched unrelated donor or 10 mg/kg BW per day for matched related donor.^32^ Thymoglobulin (Sanofi-Aventis, Paris, France) was administered at 7.5 or 4.5 mg/kg BW.^33^ For 17 patients, no information about administration of immunosuppressive antibodies was available. Donor and recipients were matched according to HLA antigens determined by PCR, as described. Related donors were available for 92 recipients (22%). For related donors, a low-resolution method, matching HLA-A, -B and DR (6/6), was used, whereas for unrelated donors, a high-resolution method, matching HLA-A, -B, -C, DQ and DR (10/10), was employed. The majority of patients were transplanted from matched donors (*n*=333; 79%), whereas 90 (21%) received stem cells from mismatched donors. For 16 male recipients, no information on donor gender was available. In our cohort, 242 (56%) recipients were male, and 33% (*n*=80) received HSCT from female donors. Six of the 429 initial patients were excluded from further analysis because of death by engraftment failure. Twenty-five patients died before day 100, six with aGvHD-complications as cause of death.

^a^For 17, no information on immunosuppressive antibodies.

^b^For 16 male recipients, no information on donor gender.

**Table 1b:** Incidence and severity of acute GvHD after allogeneic HSCT and biopsy and proteomic pattern information

	*Number of patients*	*Biopsy*	*Biospy-positive*	*aGvHD_MS17-positive*	*Biopsy-negative*	*aGvHD_MS17-negative*
aGvHDI	89	20	14	16	6	4
aGvHD II	74	21	18	11	3	10
aGvHD III	29	19	18	17	1	2
aGvHD IV	23	20	20	19	0	1
Total	215	80	70	63	10	17

The incidence and severity of acute GvHD in our patient cohort is summarized. In addition, biopsies available at time points of proteomic analyses were analyzed. Of 423 patients included in the analysis, 25 died before day +100 (aGvHD-related complications were cause of death in six patients). Acute GvHD was diagnosed in 215 patients (50%), 89 (21%) had aGvHD grade I, 74 (17.4%) and 12% (52) had severe aGvHD (aGvHD III or IV). The number of patients with biopsies (biopsy), confirmation of clinical diagnosis by biopsy (biopsy positive) or proteomic diagnostic (aGvHD_MS17-positive) and negativity of biopsy (biopsy-negative) or proteomic diagnostic (aGvHD_MS17-negative) are shown.

Twenty-five patients died before day +100 (six with aGvHD).

**Table 1c:** Acute GvHD manifestation, proteomic profiling and biopsy information

*CE-MS ID*	*ID patient*	*Age (HSCT)*	*Gender (recipient)*	*Gender (donor)*	*Overall aGvHD*	*aGvHD skin*	*aGvHD GI*	*aGvHD liver*	*aGvHD_ days HSCT*	*Sample_days post HSCT*	*aGvHD-MS17 CF*	*Biopsy_day*	*Biopsy material*	*aGVHD confirmed*	*Relapse*	*Relapse days post HSCT*	*Survival*	*Death-day HSCT*	*Cause of death*
55 931	12 173	57	w	m	I	2	0	0	41	40	−1.713	49	Skin	No (EBV-PTLD	No		No	30	EBV lymphoma
56 616	14 369	48	m	f	I	1	0	0	19	14	−0.594	20	GI	No	No		Yes		
33 018	7829	54	m	m	I	1	0	0	14	19	0.687	20	Intestine	No	No		Yes		
36 140	8429	38	w	w	I	2	0	0	28	34	−1.469	29	Intestine	No	No		Yes		
42 797	11 820	61	m	m	I	1–2	0	0	57	12	0.551	85	Intestine	No	No		Yes		
33 727	4419	59	m	w	I	2	0	0	12	4	−0.589	13	Rektum	No	Yes	453	No	653	MOF, cGvHD, lung
38 146	6194	27	w	w	0-I	0	0–1	0	35	14	0.451	35	Intestine	Yes	Yes	146	No	159	Relapse
41 229	10 765	60	m	m	I	0	0–1	0	24	22	0.582	24	Intestine	Yes	Yes	25	No	38	Relapse
33 469	3195	47	w	w	I	0	1–2	0	23	22	0.489	24	Intestine	Yes	Yes	359	No	618	Relapse
48 541	6297	36	m	m	I	2	1	0	36	27	0.441	63	Intestine	Yes	Yes	315	No	542	aGvHD, encephalopathy
36 073	8387	39	m	m	I	1–2	0	0	16	48	1.306	118	Skin	Yes	No		Yes		
44 578	5298	49	w	m	I	1–2	0	0	25	48	0.068	27	Skin	Yes	No		Yes		
36 100	8059	33	m	w	I	2	0	0	41	20	0.549	142	Intestine	Yes	No		No	202	Sepsis, MOV
35 956	8096	55	m	m	I	1	0	0	49	29	0.723	54	Intestine	Yes	No		Yes		
33 703	5384	61	m	m	I	2	0	0	9	5	0.104	50	Intestine	Yes	No		Yes		
56 514	14 371	55	w	m	I	0	1	0	16	13	−0.875	16	GI	Yes	Yes		No	150	Relapse AML
35 995	5346	49	w	w	I	1–2	0	0	34	35	−1.268	35	Skin	Yes	No		No	203	Sepsis, MOF
39 685	10 418	30	w	m	I	1	0	0	28	20	−1.35	30	Skin	Yes	No		No	66	MOV bei PTLD
37 711	9358	50	m	w	I	2	0	0	17	6	−0.911	31	Skin	Yes	No		Yes		
20 806	2719	39	m	m	I	1	0	0	48	43	−0.823	49	Skin	Yes	Yes	724	No	808	GvHD, ARDS, MOF
55 934	12 471	42	m	m	II	3	0	0	12	16	−0.261	14	GI	Negative	No		Yes		
34 491	6547	46	w	m	II	3	0	0	27	23	−1.171	139	Skin	No	No		Yes		
42 060	2046	52	m	w	II	2	yes	0	168	189	−0.388	168	Intestine	No	No		Yes		
33 022	7863	39	m	w	II	0	1–2	0	21	12	0.289	22	Intestine	Yes	No		No	130	Pneumonia or cGvHD lung
35 482	2714	37	w	w	II	0–1	2	0	22	12	0.735	24	Intestine	Yes	No		Yes		
44 597	6049	53	w	w	II	0	1–2	0	73	51	0.13	73	Intestine	Yes	Yes	55	No	144	Relapse
36 094	8039	33	w	m	II	3	1	0	24	51	0.88	65	Intestine	Yes	No		Yes		
35 836	7962	61	m	m	II	2	1	0	23	34	0.986	44	Colon	Yes	Yes	431	No	495	Cardiovascular failure, relapse
35 781	1142	47	w	m	II	2	1	0	19	16	0.322	19	GI	Yes	No		Yes		
56 470	14 229	60	w	m	II	0	1	0	22	14	0.937	12	Intestine	Yes	No		No	42	VOD, vascular complication
56 453	14 234	52	w	m	II	0	1	0	32	15	0.858	32	GI	Yes	No		No	274	EBV-PTLD
36 838	9301	35	m	n.i.	II	3	0	0	14	14	0.806	14	Skin	Yes	No		Yes		
42 096	3064	49	w	w	II	2	1	0	19	18	1.575	31	Skin	Yes	No		Yes		
36 879	8271	43	m	ni	II	2	1	0	25	17	0.348	28	Intestine	Yes	No		Yes		
45 460	12 151	33	m	m	II	3		0	13	13	−1.706	14	Skin	Yes	No		Yes		
42 570	11 359	67	m	m	II	1	1	0	27	27	−0.942	29	Skin	Yes	No		No	231	Candida sepsis, ORSA sepsis
44 587	5266	57	m	m	II	0	1	0	71	93	−1.362	75	Intestine	Yes	Yes	105	No	128	Relapse, respiratory insufficiency
56 463	14 011	55	w	f	M	2	1	0	30	14	−1.169	30	GI	Yes	No		Yes		
36 821	9297	40	m	m	II	3	0	0	23	27	−1.691	33	Skin	Yes	No		Yes		
36 825	9299	22	m	m	M	2	1	0	12	6	−0.888	18	Colon, skin	Yes	No		Yes		
56 156	13 268	59	m	f	II	2	1	0	104	105	−1.96	106	GI	Yes	No		Yes		
34 484	3344	17	m	m	III	2	2	0	16	22	0.569	92	Intestine	Yes	No		Yes		
34 903	2725	20	m	m	III	2	2	0	25	33	1.068	25	Stine (rekto/sig)	Yes	No		No	113	Respiratory failure, BO, pneumonia
37 047	8954	30	m	m	III	0	3	0	19	48	0.391	20	Intestine	Yes	No		No	432	
44 154	11 498	20	m	w	III	3	0–1	3	38	34	0.767		Liver	Yes	No		Yes		
34 486	3197	20	m	w	III	0	4	0	25	23	1.088	25	Intestine	Yes	Yes	254	No	459	Relapse
36 093	8058	58	m	m	III	1	2	0	11	5	0748	79	Intestine	Yes	No		No	92	Infection (?), MOF
41 981	11 215	67	m	m	III	2	2	0	18	20	0.074	38	Intestine	No	No		yes		
39 517	10 228	50	m	m	III	0	3	0	19	16	0.879	17	Intestine	Yes	Yes	73	No	102	Relapse
27 784	6298	45	m	w	III	1	3	0	77	43	0.227	84	Intestine	Yes	Yes	146	No	157	Relapse
35 480	2249	32	m	m	III	2	2	0	28	27	1.225	22	Intestine	Yes	No		Yes		
34 462	1695	50	m	m	III	1	4	1	36	15	1	31	Intestine	Yes	No		No	116	n.i.
49 612	10 115	50	m	f	III	0	2	0	136	133	1.024	139	Intestine	Yes	No		No	215	CNS lymphoma
56 483	14 017	56	w	m	III	1	2	0	22	7	0.738	22	Intestine	Yes	No		No	41	TTP/lung embolic comp.
55 956	14 007	56	m	m	III	0	2	0	14	79	0.523	120	Intestine	Yes	No		No	208	MOF
36 802	9290	40	w	m	III	2	0	2	26	6	0.637	0 skin 16 inte	Colon, skin	Yes	No		Alive		
35 401	6113	54	m	m	III	3	1	0	30	27	0.101	39	Skin	Yes	No		No	164	Sepsis, secondary NHL
49 229	10 922	55	w	f	III	2	2	0	10	10	0.107	16	Skin	Yes	No		Yes		
56 214	13 737	42	w	m	III	2	2	0	30	33	−1.275	34	Intestine	Yes	No		Yes		
44 582	6976	50	w	m	III	0	3–4	0	51	50	−0.459	52	Intestine	Yes	Yes	79	No	131	Relapse
56 462	14 228	45	m	m	IV	2	4	3	23	15	0.79	23	Intestine	Yes			No	175	aGvHD/MOF
55 946	12 871	48	m	m	IV	3	4	0	15	14	0.451	123	GI	Yes	No		No	241	EBV-PTLD liver
27 791	6195	55	w	w	IV	2	4	0	39	25	0.692	39	Skin	Yes	No		No	49	Septic complication
44 261	11 897	53	m	m	IV	2–3	4	0	27	27	0.57	49	Intestine	_Yes_	No		Yes		
33 019	10 447	62	m	w	IV	0	4	0	48	51	0.389	48	Intestine	Yes	no		No	129	aGvHD GI
20 867	2787	48	w	w	IV	3	yes	yes	15	22	1.048	23	Intestine	Yes	No		No	102	Septic complication
36 435	6297	37	m	w	IV	1–2	3	3	11	18	0.488	74	Intestine	Yes	No		No	119	aGvHD; MOF
36 213	8671	61	m	w	IV	3	4	2	127	51	1.039	136	Intestine	Yes	No		No	197	aGvHD
34 477	2800	50	w	w	IV	4	4	4	18	19	0.021	19	Skin	Yes	No		No	57	aGvHD, pneumonia
41 571	11 097	71	m	m	IV	0	4	yes	40	6	0.868	48	Intestine	Yes	No		No	66	aGvHD, MOF
40 555	10 743	61	w	w	IV	2	4	0	8	12	0.741	20	Intestine	Yes	No		No	125	GvHD
44 972	12 098	46	m	w	IV	1–2	4	0	18	7	0.674	22	Intestine	Yes	Yes	18	No	24	Relapse
34 269	6116	35	m	m	IV	4	4	3	54	49	0.68	54	Intestine	Yes	No		No	134	aGvHD, MOF
41 980	11 218	22	w	m	IV	2–3	4	0	14	7	0.09	37	Intestine	Yes	No		Yes		
34 857	3049	17	m	w	IV	1	4	0	29	17	0.424	31	Intestine	_Yes_	No		No	275	Intracerebral mycosis
44 589	9839	66	w	w	IV	0	4	0	51	19	0.894	52	Intestine	Yes	No		Yes		
27 792	6194	26	w	w	IV	0	4	0	23	20	0.797	23	Intestine	Yes	Yes	443	No	707	Relapse
42 669	11 620	39	w	m	IV	0	biopsy	4	27	11	1.059	27	Intestine	Yes	No		No	85	GvHD, pulmonary infection, AKF,
41 249	10 882	62	m	w	IV	3	4	0	28	19	0.152	42	Intestine	Yes	No		No	187	GvHD, hemorrhagische Zystitis
41 250	10 764	43	m	w	IV	0	4	0	36	34	−0.061	37	Intestine	Yes	No		No	147	GvHD, Sepsis

Abbreviations: aGvHD, acute graft-versus-host disease; AKF, acute kidney failure; AML, acute myeloid leukemia; ARDS, acute resiratory distress syndrom; BO, bronchiolitis obliterans; CE-MS ID, identification number of capillary electrophoresis coupled on-line to mass spectrometry analysis; cGvHD, chronic graft-versus-host disease; EBV, Ebstein-Barr virus; f, female; GI, gastrointestinal; HSCT, hematopoietic stem cell transplantation; ID patient, identification number patient; M, male; MOF, multiorgan failure; NHL, Non-Hodgkin’s lymphoma; n.i., not identified; ORSA, oxicillin resistant staphylococcus aureus; PTLD, post-transplant proliferative disorder; VOD, veno-occlusive disease; W, female.

The proteomic data of 80 patients who had biopsy information and proteomic scoring available are summarized. Identification numbers, age at HSCT and gender (recipient/donor) are shown. Incidence and severity of aGvHD ‘overall’ in different organs (skin, intestine or GI and liver) are shown. Source of biopsy material obtained is indicated. Overall grade of aGvHD and organ manifestation, as well as severity of aGvHD, is indicated. The table summarizes clinical diagnosis of aGvHD (aGvHD_days_HSCT), day of sample for the first positive proteomic pattern (sample_days post HSCT) and day of biopsy. Proteomic CF (aGvHD_MS17_CF) at the time of diagnosis (sample_days post HSCT) is indicated. ‘aGvHD confirmed’ (biopsy confirmation of aGvHD). Relapse, survival and cause of death within this group are shown.

### Urine sample collection and preparation

A volume of 10 ml of second morning midstream urine was obtained from the participants and immediately frozen at −20 °C. Samples were collected before HSCT, and on days 0 to 35 (+/−3 days) on a weekly basis and bimonthly thereafter. Sample preparation was done as previously described.^19^ A median of three samples (range 1–10) were analyzed per patient.

### CE-MS analysis and data processing

CE-MS analysis was performed as previously described^15, 16, 19, 20^ using a P/ACE MDQ (Beckman Coulter, Fullerton, CA, USA) coupled on-line to a Micro-TOF MS (Bruker Daltonic, Bremen, Germany). Mass spectral ion peaks representing identical peptides at different charge states were deconvoluted into molecular mass using MosaVisu software.^14^ Migration times and ion signal intensities (amplitude) were normalized using internal polypeptide standards.^27^ The resulting peak list characterizes each polypeptide by its molecular mass (kDa), normalized migration time (min) and normalized signal intensity. Polypeptides within different samples were considered identical if the mass deviation was <50 p.p.m., and the CE migration time deviation was <2 min.^19^

### Adaptation of the aGvHD-specific proteomic pattern and support vector machine-based cluster analysis

The training set for the aGvHD-specific pattern was published previously^19^ and expanded here. Thirty-three samples were collected from patients with biopsy-proven aGvHD grade II or higher at the time of diagnosis (range: day +4 to +79). Controls consisted of 76 time-matched samples of patients without aGvHD and without infections or relapse at the time of sampling (Supplementary Table S1). All identified discriminatory polypeptides were combined to a support vector machine (SVM) classification model using the MosaCluster software.^17^ The SVM classifier generates a dimensionless membership probability value on the basis of a patient’s peptide marker profile, termed the classification factor (CF).^19, 20^

### Statistical methods

Estimates of sensitivity and specificity were calculated based on tabulating the number of correctly classified samples in receiver operating characteristic curves and are presented as Box-and-Whisker plots of group-specific CF distributions. Only samples collected until clinical diagnosis of aGvHD were included in this evaluation. Confidence intervals (95%) were based on exact binomial calculations using MedCalc (MedCalc version 8.1.1.0 software, Mariakerke, Belgium).

Binomial logistic regression analysis was performed to determine the relationship between proteomic classification with the aGvHD_MS17 model, demographic and clinical data (Table 2).

**Table 2:** Multiparameter logistic regression analysis of demographic and clinical variables for the prediction of aGvHD grade III or IV development

*Independent variable*	*Regression coefficient* ^a^	*S.e.*	*Significance level (*P)
aGvHD_MS17 CF	0.75	0.16	<0.0001
Age	−0.02	0.01	0.050
ATG (no=0, yes=1)	−0.83	0.36	0.022
Gender of recipient (female=0, male=1)	1.23	0.31	0.0001
Gender of donor (female=0, male=1)	−0.59	0.28	0.037
Conditioning (RIC=0, myeloablative=1)	−0.69	0.38	0.05
CRP (mg/l)	−0.001	0.003	0.72
Diagnosis (acute leukemia=0, chronic leukemia=1, lymphoma=2, nonmalignant=3)	−0.45	0.23	0.046
Donor (related=0, unrelated=1)	−0.31	0.33	0.34
HLA match (matched=0, mismatched=1)	0.22	0.34	0.51
Serum albumin (g/l)	−0.06	0.05	0.07
Stage (no CR=0, CR 1/CP 1=1, CR>2=2)	0.27	0.18	0.14
Days post HSCT	−0.018	0.34	0.001

Abbreviations: aGvHD, acute graft-versus-host disease; ATG, antithymocyte globulin; CP, chronic phase; CR, complete remission; CRP, C-reactive protein; HLA, human leukocyte antigen; HSCT, hematopoietic stem cell transplantation; RIC, reduced intensity conditioning regimen.

Multiparameter, logistic regression analysis is shown to determine the relationship between proteomic classification with the aGvHD_MS17 model, demographic and clinical data as predictor variables for development of severe aGvHD grades III and IV. Clinical data, such as age and gender of the patient and donor, conditioning regimen (RIC or standard), presence or absence of immunosuppressive antibodies (ATG or thymoglobulin), primary disease, stage of disease before HSCT, related or unrelated donors, HLA-matching of donor and recipient, levels of serum albumin (g/l)^21^ and CRP (mg/l)^22^ were used in this model.

^a^Expresses the amount of change in the logit function related to one unit change in the predictor.

### Peptide sequencing

Urine samples were analyzed on a Dionex Ultimate 3000 RSLS nano flow system (Dionex, Camberly, UK) as described previously.^19^ All polypeptides forming aGvHD_MS17 are shown with their CE-MS characteristics (Table 3) and sequences. More detailed information and additional data can be found in the Supplementary Material provided at the journal’s website.

**Table 3:** Characteristics of urine peptides forming the aGvHD_MS17 pattern

			*Peptide distribution in the training cohort*								
*CE-MS characteristics*			*No GvHD (*n*=57)*		*GvHD grade I (*n*=19)*		*GvHD grade II–IV (*n*=35)*		*Sequence information*		
*Peptide ID* ^a^	*CE migration time (min)*	*Mass (Da)*	*Mean amp*	*Freq*	*Mean amp*	*Freq*	*Mean amp*	*Freq*	*Sequence* ^b^	*Protein name*	*AA* ^c^
3696	21.54	882.4	77	0.52	69	0.51	162	0.71	n.i.		
23 968	36.18	1191.5	152	0.50	88	0.38	71	0.27	pPGSNGNpGPpGP	Collagen a-1(II) chain	907–919
30 177	21.42	1292.6	62	0.23	71	0.26	17	0.08	n.i.		
45 503	39.98	1540.8	831	0.63	944	0.74	1456	0.79	GPpGVPGpPGpGGSPGLP	Collagen a-1 (XXII) chain	717–734
82 094	19.84	2228.1	815	0.15	479	0.30	1697	0.59	DAHKSEVAHRFKDLGEENF	Serum albumin; N-term.	25–43
84 126	33.55	2257.0	552	0.70	299	0.49	583	0.67	QG PAG EpG EpGQTG PAGARG PAG pP	Collagen a-2(I) chain	114–138
100 537	20.07	2603.3	6281	0.27	6810	0.40	17 274	0.63	LKNGERIEKVEHSDLSFSKDWS	P-2-microglobulin	60–81
105 836	23.38	2708.3	183	0.22	339	0.38	942	0.67	KGQpGApGVKGEpGApGENGTpGQTGARG	Collagen a-2(I) chain	189–217
110 841	23.71	2821.3	247	0.38	369	0.53	763	0.71	LkGQpGApGVKGEpGApGENGTPGQTGARG	Collagen a-2(I) chain	188–217
118 597	23.42	3021.4	611	0.71	247	0.51	202	0.29	DGVSGGEGKGGSDGGGSHRKEGEEADAPGVIPG	CD99 antigen	97–129
119 142	24.93	3033.4	94	0.23	329	0.30	408	0.36	LDGAKGDAGPAGPKGEpGSpGENGApGQMGPRG	Collagen a-1 (I) chain	273–305
119 538	29.98	3041.4	1979	0.94	1664	0.91	928	0.69	DGIHELFPAPDGEEDTAELQGLRPGSEY	Fibronectin	1671–1698
133 508	22.69	3443.6	155	0.10	249	0.21	1076	0.49	n.i.		
145 889	24.53	3891.8	487	0.50	454	0.32	134	0.13	n.i.		
148 384	19.48	3995.9	185	0.10	197	0.17	1533	0.37	n.i.		
160 240	23.00	4441.0	368	0.10	304	0.13	1475	0.43	n.i.		
164 539	23.12	4613.1	307	0.10	544	0.23	2154	0.57	n.i.		

Abbreviations: AA, amino acid; amp, amplitude; CE-MS, capillary electrophoresis coupled on-line to mass spectrometry; GvHD, graft-versus-host disease; Freq, frequency; n.i., not identified.

The table gives the peptide identification number (Peptide-ID), experimental mass (in Da) and CE migration time (in min) for all 17 peptides included the urinary aGvHD_MS17 peptide marker model. For all sequence-identified peptides, the AA sequence, the name of the protein precursor and the AA positions within the protein’s primary sequence (according to UniProtKB) are presented. In addition, the frequency and the mean amplitude in the number of GvHD, GvHD grade I and GvHD grade II–IV groups of the training cohort are provided.

^a^Peptide identification numbers.

^b^Hydroxylation of proline and lysine is indicated in the amino acid sequence by lower case ‘p’ and ‘k, respectively.

^c^Positions of first and last AA according to UniProt Knowledge Base numbering.

## Results

### Patient characteristics

In this prospective validation study, 423 patients from five transplant centers were evaluated with the aGvHD-specific aGvHD_MS17 peptide marker pattern. A summary of relevant clinical data is shown in Table 1a and described in Methods. Table 1b lists the incidence and severity of aGvHD and gives information on biopsies obtained within our cohort. Acute GvHD developed in 215 patients (50%). Grade I was diagnosed in 21.5% (*n*=89), whereas 17.5% (*n*=74) had aGvHD grade II. Twelve percent (*n*=52) of the patients developed aGvHD III (*n*=29) or IV (*n*=23) despite GvHD prophylaxis and additional immunosuppressive antibodies (antithymocyte globulin) (Table 1b). Biopsy results and proteome analysis at the same time point were available from 80 patients. aGvHD was histologically confirmed in 70 patients. Of those, 32 had aGvHD grade I or II and 38 had GvHD grade III or IV. Only the latter were included to the in-depth analysis. Diagnosis based on biopsy and proteomic profiling is compared in Table 1b. Table 1c summarizes the data of biopsies and aGvHD-MS17 diagnostics.

### Proteomic patterns (aGvHD_MS17) for aGvHD assessment

**Figure 1:**
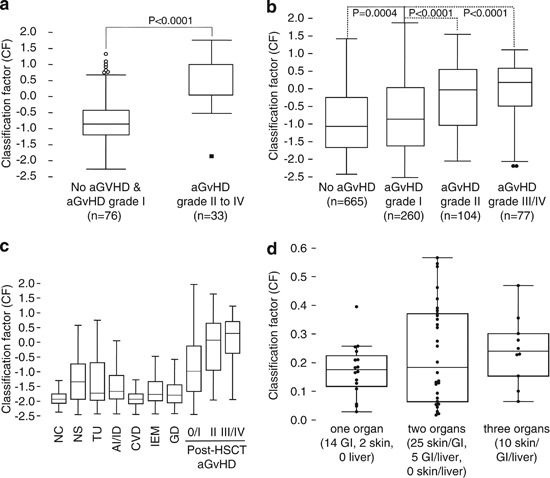
Patients and samples in the model establishment and prospective evaluation phase. (**a**) Distribution of the CF in the training set. Box-and-Whisker plot presentation showing the difference in aGvHD_MS17 classification between patients with aGvHD grade II or more compared with the controls for the training set. The training set consists of 33 samples with aGvHD grade II or more, and 76 samples from control patients. The pattern was transformed into a CF shown on the *y* axis using MosaCluster, an SVM-based program. MosaCluster constructs a separation hyperplane between the case and control samples of the training set in the *n*-dimensional aGvHD biomarker space. The result of SVM classification is a dimensionless positive or negative number termed as CF representing the Euclidian distance of a sample data point to the constructed separation hyperplane. The CF with the best sensitivity–specificity ratio in receiver operating characteristic evaluation of SVM values of the training set was defined as the cut-off point, in this case CF ⩾0.1, and used subsequently as decision criterion for aGvHD prediction in all prospectively collected samples. (**b**) Distribution of the CF in the prospective samples (*n*=1106). Comparison of aGvHD_MS17 CF values in the prospective HSCT patient cohort for the differentiation of aGvHD grade I from grade II and >grade II. All samples of the prospective cohort were analyzed and correlated with the clinical data. Box-and-Whisker representation of group-specific CF distribution is shown for the groups ‘no GvHD’, ‘aGvHD grade I’, ‘aGvHD grade II’ and ‘aGvHD grade III/IV’ of the prospective validation cohort (423 patients, 1106 samples) until clinical diagnosis of aGvHD. For the calculation of *P*-values, a *post-hoc* rank test was performed for average rank differences between the aGvHD grade I reference group and the aGvHD grade II and >grade II case groups after a significant result in the global Kruskal–Wallis test (*P*<0.0001). (**c**) Specificity of aGvHD_MS17. Comparative analysis of aGvHD_MS17 model classification of samples collected from: NC, normal controls (*n*=76); NS, patients with nephrotic syndromes (*n*=253) including minimal change disease (*n*=12), focal segmental glomerulosclerosis (*n*=106), membranous glomerulonephritis (*n*=55), membranoproliferative glomerulonephritis (*n*=4) and IgA nephropathy (*n*=76); CVD, patients with cardiovascular diseases (*n*=234) including myocardial infarction (*n*=87), atherosclerosis (*n*=7), hypertension (*n*=45) and coronary disease (*n*=95); TU, patients with tumors (*n*=160) including Kaposi’s sarcoma (*n*=68), pancreatic carcinoma (*n*=11), cholangiocarcinoma (*n*=68), hepatocellular carcinoma (*n*=9) and tumors of other origin (*n*=4); IEM, patients with inborn error of metabolism (*n*=239) including type 2 diabetes mellitus (*n*=78) and Fabry disease (*n*=161); AI/ID, patients with autoimmune or inflammatory disorders (*n*=661) including type 1 diabetes mellitus (*n*=503), systemic lupus erythematosus (*n*=18), cholestasis (*n*=115) and vasculitis (*n*=25); GD, patients with genetic diseases (*n*=118) including autosomal-dominant polycystic kidney disease (*n*=71) and polycystic ovary syndrome (*n*=47). These non-disease-related control groups were compared with samples collected from patients after allo-HSCT without aGvHD or aGvHD grade I, aGvHD grade II or aGvHD III and IV. (**d**) Organ involvement in severe aGvHD. Figure 1d shows the Box-and-Whisker analyses of aGvHD_MS17 scoring for organ involvement in severe aGvHD. Applying proteomic profiling does not describe involvement of particular organs; however, severity of aGvHD is usually also accompanied by more than one organ manifestation. Manifestation of aGvHD in specific organs is indicated. GI, gastrointestinal manifestation.

The aGvHD_MS17 proteomic classifier was designed to predict patients at risk for development of severe aGvHD. Quantitative differences in the excretion of the pattern-forming peptides were observed upon comparison of patients without aGvHD, patients with aGvHD grade I and those with biopsy-proven aGvHD grade II or more sampled at clinical diagnosis of aGvHD (Table 2). The differences in the excretion of the peptides included in the proteomic classification model aGvHD_MS17 were converted to a numerical CF, using an SVM-based clustering software as described.^19^ Box-and-Whisker plot analysis of CF values in the case and control patient groups of the training set (Supplementary Table S1) demonstrated a significant difference of the aGvHD_MS17 classifier in samples from patients without aGvHD or aGvHD grade I (*P*<0.0001) when compared with patients with aGvHD grade II or more (Figure 1a). Analyses of 1106 samples collected from our prospective cohort provided further evidence that the proteome classifier aGvHD_MS17 can significantly distinguish patients with no aGvHD from those with aGvHD grade I (*P*=0.0004), grade II (*P*<0.0001) or grades III/IV (*P*<0.0001), respectively (Figure 1b). To evaluate the specificity of aGvHD_MS17, additional control samples including chronic renal failure syndromes and autoimmune diseases were analyzed with the same classifier as patients after allo-HSCT (Figure 1c). Only samples from patients after allo-HSCT with severe aGvHD were positive in aGvHD_MS17 classification. Organ manifestation of aGvHD was analyzed in the prospective set for prediction of organ involvement. aGvHD_MS17 scoring was investigated for skin, intestine or liver manifestation of aGvHD to examine possible organ-specific effects on the classification. Although no significant difference between the different manifestations could be detected (data not shown), indicating absence of organ specificity of aGvHD_MS17, involvement of more than 1 organ, which usually correlated with a higher grade of aGvHD, resulted in higher CF values (Figure 1d), as expected.

### Peptides and proteins forming the aGvHD_MS17 proteomic pattern

To date, we have successfully sequenced 10 of 17 pattern-forming, naive peptides. In patients with aGvHD, we found increased excretion of fragments of albumin (N-terminal), β2-microglobulin, collagen-α1 and -α2, and decreased excretion of fragments of CD99, fibronectin and collagen-α1 (Table 3).

### Multivariable logistic regression and receiver operating characteristic analysis

Consecutive logistic regression analysis using aGvHD grade III or IV onset 14 days before any clinical signs for aGvHD as a dependent binary variable (Methods and Table 2) demonstrated that positivity in the aGvHD_MS17 model was the strongest predicting variable (*P*<0.0001) for the development of severe aGvHD. Recipient gender (*P*=0.0001) was also a highly significant predictor in our cohort (Table 2), with a predisposition of aGvHD development in males. Donor gender (*P*=0.037) was also a significant variable; male recipients transplanted from female donors had the highest risk for aGvHD development. Other significant variables were age, conditioning (*P*=0.05), immunosuppressive antibodies (*P*=0.02), primary disease (acute myeloid leukemia; *P*=0.046) and days post HSCT (*P*=0.001). C-reactive protein and serum albumin did not correlate with aGvHD development (*P*-values of 0.72 and 0.07, respectively) and therefore did not improve classification performance of the logistic regression model.

**Figure 2:**
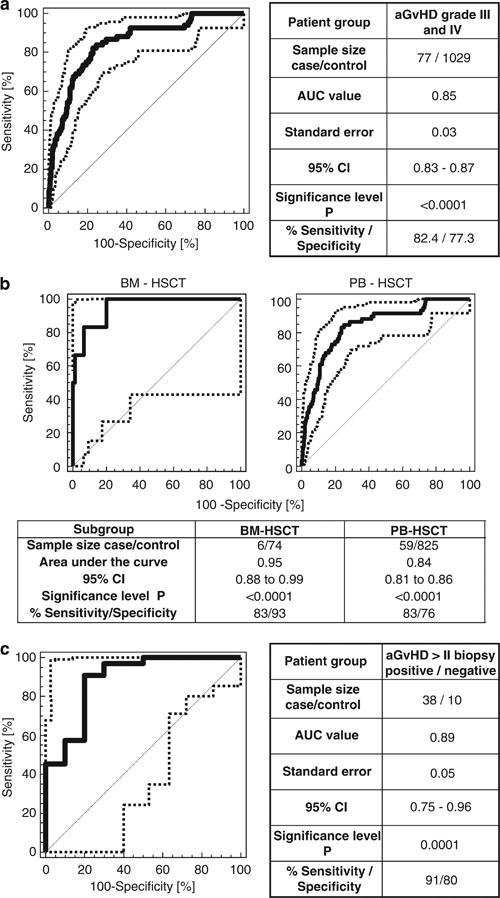
(**a**) Prediction of severe aGvHD 14 days before clinical signs in the prospective patient cohort. Receiver operating characteristic (ROC) curve (bold line, area under the curve (AUC)=0.85) of aGvHD grade III/IV prediction 14 days before any signs of aGvHD by the logistic regression model that was generated by combining proteomic pattern diagnosis with statistically significant demographic and medical variables such as age, immunosuppressive antibodies (antithymocyte globulin/thymoglobulin) recipient and donor gender, conditioning regimen, primary disease, human leukocyte antigen-match of donor and recipient and days post HSCT. Samples taken under steroid therapy were excluded to prevent confounding effects of steroids of the blinded set (Tables 1a–c, Supplementary Table 1). 95% Confidence intervals (95% CIs) are indicated by thin, broken lines. (**b**) Prediction of aGvHD grade II or more: BM-HSCT versus PB-HSCT. Separate analyses of samples collected from 39 patients after allogeneic BM and 379 patients after PB stem cell HSCT are shown. Only samples of patients with information on all clinical and demographic variables were analyzed. Cord blood SCT recipients (*n*=5) were excluded from this analysis. Pending severe aGvHD was analyzed by application of aGvHD_MS17 positivity in combination with statistically significant demographic and medical variables. The resulting ROC curve is compared with that of patients after PB-HSCT. The AUCs (0.95 and 0.84, respectively) are shown by the bold line, and 95% CIs are indicted by dotted lines. (**c**) Biopsy-proven aGvHD: correlation to prediction of aGvHD by proteomic profiling. Biopsies of the suspected organ were available in 80 patients. In 10 cases, aGvHD was not confirmed by biopsy (control). Only patients with biopsy-confirmed aGvHD grades III/IV were included in the analysis. The correlation of aGvHD_MS17 prediction of pending aGvHD with the later biopsy-confirmed aGvHD is shown here. AUC (0.89) and 95% CI are shown.

A logistic regression model combining the aGvHD_MS17 CF values with the statistically significant demographic and clinical variables presented in Table 2 enabled diagnosis of severe aGvHD with a sensitivity of 82.4% and a specificity of 77.3% about 14 days before clinical diagnosis and at a time when the patients had no clinical signs of aGvHD (Figure 2a). CF of 0.1 was determined as the most discriminatory cut off. Separate analyses of recipients of bone marrow (BM) grafts (*n*=39) revealed high sensitivity (83%) and specificity (93%) for prediction of severe aGvHD development (Figure 2b). In addition, we compared the proteomics data with data obtained from biopsies where available. Figure 2c shows the receiver operating characteristic for both diagnostic tools in comparison. The prediction of severe aGvHD by aGvHD_MS17 proteomic profiling is comparable to the diagnosis based on biopsies (Table 1c, Figure 2c). Patients with biopsy-proven aGvHD grade III/IV were predicted correctly with aGvHD_MS17 with 91% sensitivity and 80% specificity. In addition, positivity of aGvHD_MS17 was usually detected earlier than positivity in biopsies (Table 1c, Figure 2c).

To test the ability of the aGvHD_MS17 pattern to discriminate between aGvHD and cGvHD, we evaluated samples from patients with manifested cGvHD and samples collected after day +130 post HSCT upon complete withdrawal of immunosuppression. The aGvHD_MS17 pattern did not cross-react with patients with manifested cGvHD (Supplementary Figure S1). Late-onset aGvHD upon withdrawal of immunosuppression was diagnosed using aGvHD_MS17 and presented as ‘aGvHD’ in our biomarker panel. The data demonstrate that the combination of aGvHD_MS17 with relevant demographic and medical variables provides for the first time the opportunity for preemptive treatment of patients at risk for severe aGvHD.

## Discussion

Evaluation of the aGvHD-specific proteomic pattern aGvHD_MS17 over a period of 5 years in five different transplant centers demonstrated its power to predict aGvHD and potential usefulness to select patients for preemptive therapy. Blinded samples were classified correctly, with a sensitivity of 82.4% (95% confidence interval: 71–92.4) and specificity of 77.3% (95% confidence interval: 73.7–79.2) in combination with demographic and medical variables using a logistic regression model (Figure 2). Separate analyses of samples from patients after BM or peripheral blood (PB) stem cell transplantation showed that the performance of aGvHD_MS17 was statistically significantly better (*P*=0.01) in patients after BM-HSCT (area under the curve: 0.95). The sensitivity and specificity were 83% and 93% compared with 83% and 76%, respectively, in the PB-HSCT (area under the curve: 0.84) recipients. However, only 39 patients received BM-HSCT grafts, whereas 379 received PB-HSCT grafts.

Importantly, the aGvHD_MS17 is specific for prediction of aGvHD, especially grades III and IV, and does not cross-react with patients with other diseases or complications tested (Figure 1) or samples from patients with cGvHD (Supplementary Figure S1). In addition, aGvHD_MS17 positivity was the most significant independent variable in the multivariable logistic regression model, predicting development of aGvHD grades III and IV, followed by gender, whereas conditioning regimen and even matched donor transplantation were less significant (Table 2).

The loss of serum albumin in patients developing aGvHD grades III and IV of the intestine has been described recently, leading the authors to speculate that albumin might be lost via the intestine as aGvHD-initiated organ damage progresses.^21^ The majority of patients had decreased albumin levels early after HSCT; however, inclusion of serum albumin levels in our multivariate regression model showed that serum albumin loss was not statistically significant in our cohort for prediction of severe aGvHD. The decreased serum albumin levels observed in our study may have resulted from the administration of immunosuppressive antibodies to 72% of our patients during conditioning (Tables 1a–c). Capillary leakage syndromes are common under this conditioning therapy and may be the underlying cause of serum albumin loss in our patients independent of aGvHD. However, we detected increased urinary excretion of a specific N-terminal fragment of albumin as aGvHD progressed (Table 3). Albumin uptake in T cells was described to be associated with aGvHD development.^28^ Thus, our results confirm those of Rezvani *et al.*,^21^ but suggest changes in serum albumin metabolism/catabolism or possible GvHD-induced vascular damage in the kidney rather than mere intestinal loss of serum albumin as a pathological component of aGvHD.

Others have applied new technologies for aGvHD diagnosis, underlining the need for advances in the ability to diagnose GvHD in patients undergoing allogeneic HSCT.^23, 29, 30^ A biomarker panel consisting of six proteins potentially involved in the pathogenesis of aGvHD (IL-2 receptor-α, tumor necrosis factor receptor-1, hepatocyte growth factor, IL-8, elafin, a skin-specific marker,^23^ and regenerating islet-derived 3-α)^31^ was established for serum using enzyme-linked immunosorbent assay. These biomarkers, present at the time of diagnosis of manifested aGvHD, were investigated in a multicenter trial to predict treatment response and survival of patients with aGvHD.^30^ Sampling was done at diagnosis of manifested aGvHD and 14 and 28 days after initiation of treatment, and the pattern could predict response to therapy and survival. However, these markers are not suitable for preemptive diagnosis of aGvHD.^30^ The special value of our aGvHD-specific classifier (aGvHD_MS17) is its capacity to identify patients before any clinical signs of developing aGvHD, independent of organ manifestation and at least 14 days before clinical manifestation of aGvHD. The aGvHD_MS17 classifier is in very good agreement with the gold standard for aGvHD diagnosis, namely tissue biopsies (Tables 1a–c, Figure 2d). Tissue biopsy cannot be used for routine monitoring requiring repeated sampling, and its predictive value is therefore not easily assessable. Prediction of pending severe aGvHD can currently only be accomplished by the proteomic pattern. No association of specific organ manifestations of aGvHD was detectable. However, the severity of pending aGvHD, as well as manifestation of aGvHD in more than one organ, was both associated with aGvHD_MS17 scoring. In our cohort, patients with severe aGvHD had generally more than one organ involved in aGvHD, as well as a higher score in the aGvHD_MS17 classifier (Figure 1d).

Sequencing the naive peptides forming the classifier (aGvHD_MS17) provided insight into aGvHD pathophysiology and, ultimately, may help to identify novel potential therapeutic targets for aGvHD therapy. We observed increased or decreased excretion of the pattern-forming peptides. For example, increased β2-microglobulin excretion may indicate cell death as aGvHD progresses in severity. In addition, we observed increased or decreased excretion of particular collagen fragments, indicating very early changes in collagen metabolism, possibly indicating inflammation and/or early vascular damage that may consequently lead to organ damage. It is well accepted that conditioning, especially with total body irradiation, leads to an inflammatory environment, which causes activation of recipient antigen-presenting cells and donor T cells. CD99, for example, is an activation marker of T cells, and excretion was decreased as aGvHD severity increased. One can speculate that in the activation state (aGvHD) turnover of CD99 may be reduced. Interestingly, the decreased excretion of the fibrinogen fragment points toward unsuccessful repair of the microdamages to the vasculature in patients prone to develop aGvHD III/IV (Table 3).

In summary, application of the proteomic classifier (aGvHD-MS17) to evaluate allo-HSCT recipients allowed reliable prediction of specific changes and damages relevant for our understanding of aGvHD development. Urinary proteomic monitoring introduces the first unbiased, investigator-independent diagnosis of pending severe aGvHD and are currently investigated to guide preemptive treatment of aGvHD_MS17 pattern-positive patients in clinical trials.

## Supplementary information

Supplementary Figure 1 (PPT 78 kb)

Supplementary Tables (XLS 52 kb)

Supplementary Information (DOC 34 kb)
