# Supplementary material for: Proteomic peptide profiling for preemptive diagnosis of acute graft-versus-host disease after allogeneic stem cell transplantation
Source: Leukemia. 2013 Jul 11;28(4):842–52. doi: 10.1038/leu.2013.210 (PMC7101954; doi:10.1038/leu.2013.210)
Supplement: Supplementary file 1 — Supplementary Figure 1 (PPT 78 kb) [file 41375_2014_BFleu2013210_MOESM11_ESM.ppt]

## Slide 1
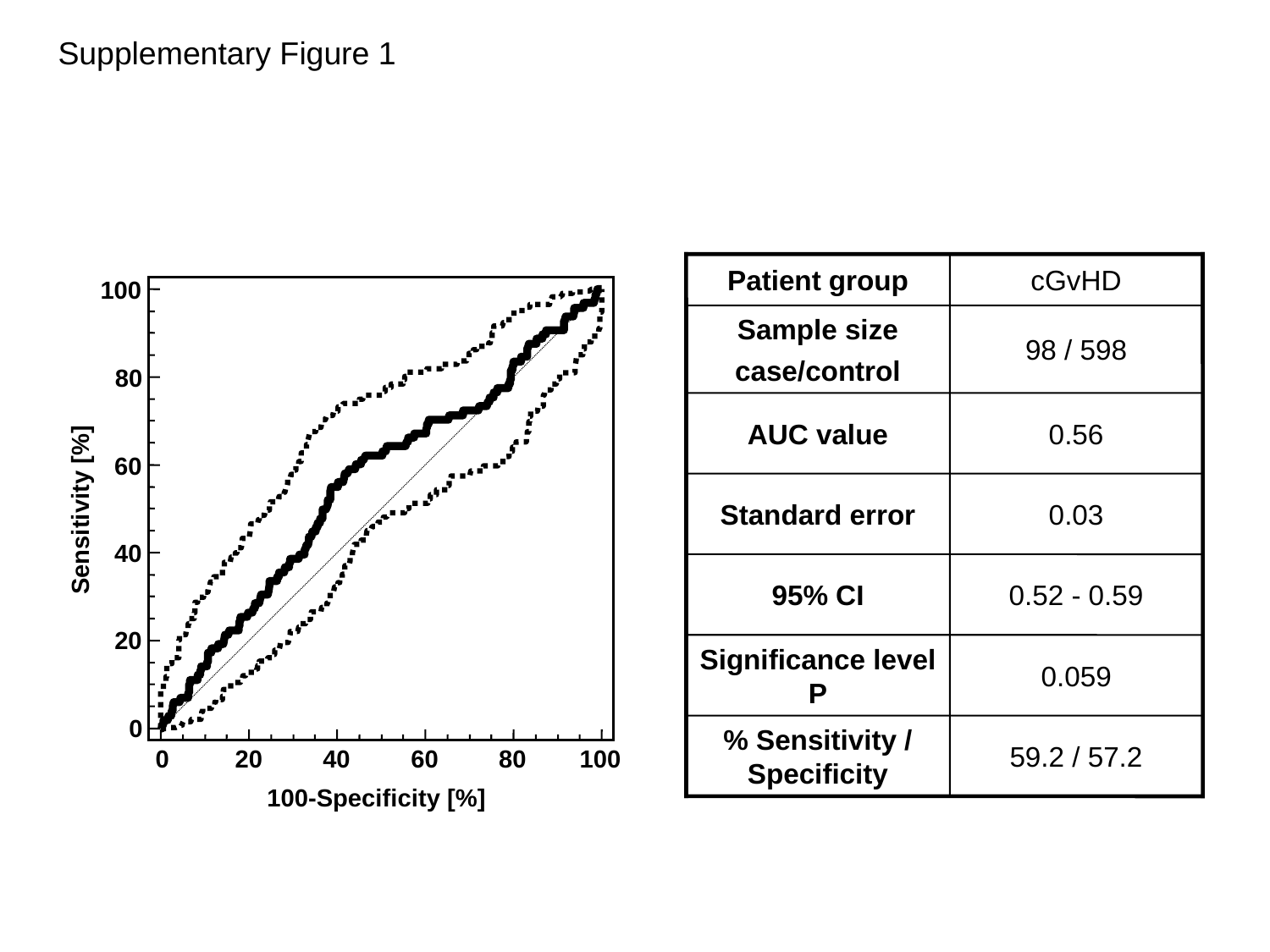

Supplementary Figure 1
Patient group
cGvHD
Sample size
case/control
98 / 598
AUC value
0.56
Standard error
0.03
95% CI
0.52 - 0.59
Significance level P
0.059
% Sensitivity / Specificity
59.2 / 57.2
100
80
60
Sensitivity [%]
40
20
0
0
20
40
60
80
100
100-Specificity [%]
