# Supplementary material for: Proteomic peptide profiling for preemptive diagnosis of acute graft-versus-host disease after allogeneic stem cell transplantation
Source: Leukemia. 2013 Jul 11;28(4):842–52. doi: 10.1038/leu.2013.210 (PMC7101954; doi:10.1038/leu.2013.210)
Supplement: Supplementary file 3 — Supplementary Information (DOC 34 kb) [file 41375_2014_BFleu2013210_MOESM13_ESM.doc]

**Sample preparation:**

Freshly thawed urine (0.7 ml) was diluted (1:2) with an aqueous solution of 2 M urea, 10 mM NH_4_OH and 0.02 % SDS. Proteins larger than 20 kDa molecular weight were removed by filtration through Centrisart ultracentrifugation devices (Sartorius, Göttingen, Germany) at 3,000 g until 1.1 ml of filtrate was obtained. The filtrate was desalted with a PD-10 column (GE Healthcare, Munich, Germany) pre-equilibrated with 0.01% NH_4_OH in HPLC-grade H_2_O to decrease matrix effects by urea, electrolytes and salts. After elution, samples were lyophilized and re-suspended in CE-MS running buffer containing 20% acetonitrile and 1% formic acid to a final protein concentration of 0.8 µg/µl based on the bicinchoninic acid assay (Interchim, Montlucon, France) directly before CE-MS analysis. For acute GvHD development, samples collected up to day +130 (+/-30) post-allo-HSCT were analyzed as indicated by the solid horizontal arrow. To evaluate aGvHD_MS17 cross reactivity of patients with cGvHD, samples collected after day +130 were analyzed.

**CE-MS analysis:**

After separation in the CE, peptides were ionized and sprayed directly into the MS. The ESI sprayer (Electro-spray-ionization; Agilent Technologies, Palo Alto, CA, USA) was grounded, and the ion spray interface potential was set between –3 and –4 kV. Data acquisition and MS acquisition methods were automatically controlled by the CE via contact-close-relays. Spectra were accumulated every 3 s, over a mass/charge (m/z) range of 350 to 3000.

**Sequencing of pattern forming peptides**

Briefly, elution was performed on an Acclaim PepMap C18 nano column 75μm x 15cm, 2μm 100 Å with a linear gradient of 0.1% formic acid as solvent A against 100% acetonitrile as solvent B starting at 5% to 50% B over 100 min. The sample was subsequently ionized in positive ion mode using a Proxeon nano spray ESI source (Thermo Fisher Hemel UK) and analyzed in an Orbitrap Velos FTMS (Thermo Finnigan, Bremen, Germany). The MS was operated in data-dependent mode to automatically switch between MS and MS/MS acquisition and parent ions were fragmented by HCD. Data files were searched against the IPI human non-redundant database using the Open Mass Spectrometry Search Algorithm (OMSSA, <http://pubchem.ncbi.nlm.nih.gov/omssa>) without any enzyme specificity. Mass error windows of 10 ppm and 0.05 Da were allowed for MS and MS/MS, respectively. The correlation between peptide charge at the working pH of 2 and CE-migration time was used to minimize false-positive identification rates.^16,27^ Table 3 shows all 17 pattern-forming peptides and their relevant data.

**Supplementary Figure 1: aGvHD_MS17 does not cross react with samples of patients with cGvHD**

The classifier aGvHD_MS17 was used to evaluate samples collected from patients after day +130 who were off immunosuppression or who had been diagnosed with chronic GvHD based on clinical parameters. The ROC curve indicates that aGvHD_MS17 does not cross react with samples from patients with chronic GvHD. The discriminator is indicated by a bold line, while the 95% CI is shown by fine lines.

**Table S1: Clinical and demographic data of all patients:**

Abbreviations: ID: identification number; CE/MS: capillary electrophoresis / mass spectrometry; Pat: patientAM(L)L: acute myeloid (lymphatic) leukemia; CM(L)L: chronic myelogenoous (lymphatic) leukemia; sAML: secondary AML HD: Hodgkin disease; NHL: non-Hodgkin lymphoma; foll: follicular; GI : gastrointestinal; MDS: myelodysplastic syndrome; MM: multiple myeloma; (V)SAA very severe aplastic anemia; CR: complete remission; CP: chronic phase; PR: partial remission; PD: progressive disease; HLA-match: 1: HLA-identical2: mismatch; MRD: matched related donor; MUD: matched unrelated donor; mMUD: mismatched unrelated donor: mMRD mismatched related donor; SYN: syngeneic donor Bu: busulfan Cy: cyclophosphamid; TBI: total body irradiation (12 Gy) or TBI8 : 8Gy or as indicated; TLI total lymphoid irradiation; TCD: T-cell depletion ex vivo; ATG anti-thymocyte globulin; Thymo: thymoglobulin Flu: fludarabine (F); RIT: radioimmunotherapy (zevalin); VP16: vincristin; ICE: idarubicin cyclophospamid etoprosid; ARAC: AMSA: amsacrin; FLAMSA: fludarabin, amsacrin, cyclphosphamid, TBI (4GY) or Bulsilvex (as indicated); CVB: cyclophosphamid/etoposid/BCNU, FBM: fludarabin, carmustine (BCNU) and melphalan; BAC: busulfan, cytosine arabinoside, and cyclophosphamide RIC: reduced intensity conditioning; CSA: cyclosporin A; MTX: methotrexat; MMF: mycophenolate mofetil; pred: prednisolon MOV: multiorgan failure; VOD venoocclusive disease; ANV: acute renal failure n.i. no information
